# Impact of rapid pathogen identification of gram-negative anaerobic bacteria from blood cultures on antimicrobial stewardship

**DOI:** 10.1128/spectrum.02383-25

**Published:** 2025-10-31

**Authors:** Nour Khankan, Thomas J. Rust, Andrea M. Pallotta, Stephanie N. Bass, Christine Ramsey, Bethany Burns, Daniel D. Rhoads, ChungYun Kim

**Affiliations:** 1 Department of Pharmacy, Cleveland Clinic 2569, Cleveland, Ohio, USA; 2 Department of Pathology and Laboratory Medicine, Cleveland Clinic 2569, Cleveland, Ohio, USA; 3 Department of Pathology, Cleveland Clinic Lerner College of Medicine, Case Western Reserve University 242911, Cleveland, Ohio, USA; 4 Infection Biology Department, Lerner Research Institute, Cleveland Clinic 22516 , Cleveland, Ohio, USA; Universita degli Studi dell'Insubria, Varese, Italy

**Keywords:** rapid pathogen identification, antimicrobial stewardship, positive blood culture broth, Gram-negative anaerobic bacteria, bloodstream infection, MALDI-TOF MS

## Abstract

Anaerobic bacteria are an important but understudied cause of bacteremia. The objective of this study was to evaluate the impact of using matrix-assisted laser desorption/ionization time-of-flight mass spectrometry (MALDI-TOF MS) to rapidly identify obligate anaerobic gram-negative bacteria directly from positive blood culture broth (PBCB) samples. This retrospective pre- and post-intervention study included patients with blood cultures positive for anaerobic gram-negative organisms, comparing outcomes before and after the implementation of a direct-from-PBCB MALDI-TOF MS workflow. The primary outcome was the time from Gram stain to organism identification. A secondary outcome was the time to first antibiotic modification (escalation or de-escalation). In monomicrobial anaerobic bloodstream infections, the median (interquartile range [IQR]) time from Gram stain to pathogen identification was significantly reduced in the post-intervention group compared to the pre-intervention group (15.0 hours [IQR: 7.8–47.8] vs 45.3 hours [IQR: 41.5–49.3]; *P* < 0.001). Similarly, the median (IQR) time to the first antibiotic de-escalation was significantly shorter following implementation of directly-from-PBCB MALDI-TOF MS (21.1 hours [IQR: 7.9–51.6] vs 52.0 hours [IQR: 24.0–64.5]; *P* = 0.014). This study demonstrates that the rapid identification of gram-negative obligate anaerobes from PBCB can help to significantly expedite antibiotic therapy de-escalation.

**IMPORTANCE:**

Obligate anaerobic gram-negative organisms are a significant but understudied cause of bloodstream infections. This study demonstrates that rapid identification of anaerobic gram-negative bacteria directly-from-positive blood culture broth by matrix-assisted laser desorption/ionization time-of-flight mass spectrometry can substantially reduce the time to both pathogen identification and antibiotic optimization in monomicrobial bloodstream infections. Faster diagnostic turnaround supports more timely and targeted therapy, with important implications for antimicrobial stewardship and patient outcomes.

## INTRODUCTION

Bloodstream infection (BSI) caused by anaerobic gram-negative bacteria can be a life-threatening condition and may be indicative of a polymicrobial infection given their natural environment and infectious source (1). In order to prevent mortality and longer hospital length of stay (LOS) in patients with gram-negative anaerobic BSI, rapid pathogen identification and optimal antimicrobial therapy are beneficial (2–4). Mortality in patients with gram-negative bacteremia is reduced when the initial antimicrobial treatment effectively targets the causative pathogen (4).

Traditional anaerobic bacterial identification methods (e.g., subculture on nutrient agar) may take several days largely due to the slow-growing nature of anaerobes (5). The use of rapid diagnostic tests (RDTs) to identify the causative pathogen in bacteremia may enable earlier optimization of antimicrobial therapy (6).

Matrix-assisted laser desorption/ionization time-of-flight mass spectrometry (MALDI-TOF-MS) is a qualitative *in vitro* diagnostic device intended for rapid pathogen identification. Currently, MALDI-TOF is widely used and recognized as a strong alternative to traditional biochemical and phenotypic methods for identifying anaerobic bacteria. By analyzing the mass spectrum generated from laser ionization of a sample and comparing it with the spectra in the reference database, MALDI-TOF is able to produce an accurate pathogen identification from most samples in as early as minutes (7). Methods have also been developed to use MALDI-TOF directly from positive blood culture broth (PBCB) (8). The use of MALDI-TOF and other RDTs including rapid susceptibility testing and molecular identification methods to expedite PBCB testing remains an area of active research in clinical microbiology (9–12). However, existing literature on using RDTs for PBCB samples has primarily evaluated the impact on aerobic gram-negative bacilli and gram-positive cocci. Although several studies suggest a mortality benefit with faster time to appropriate antimicrobial coverage with molecular rapid diagnostic testing, these studies are not necessarily powered to specifically demonstrate this effect in cases of anaerobic gram-negative bacteremia (13). The current study evaluated the impact of using direct from PBCB MALDI-TOF rapid organism identification on cases of anaerobic gram-negative bacteremia.

## MATERIALS AND METHODS

The study protocol was approved by the Cleveland Clinic Health System (CCHS) institutional review board (IRB), which waived the requirement for ethical approval due to the retrospective nature of the study. This retrospective, pre-/post-intervention study was conducted across 14 sites within the CCHS, including a 1,400-bed academic medical center and 13 smaller regional community hospitals. To evaluate the turnaround time for anaerobic gram-negative pathogen identification, all blood cultures processed at the Cleveland Clinic Main Campus microbiology laboratory with Gram stain results indicating gram-negative bacilli or gram-variable bacilli—later identified by culture or MALDI-TOF to the genus or species level as an anaerobic gram-negative bacillus—were included. The pre-intervention group encompassed specimens collected between 1 January 2019 and 1 August 2021, and the post-intervention group included those collected from 1 January 2022, to 1 August 2024. To assess the time to antimicrobial modification, all adult patients (aged ≥18 years) admitted to CCHS with blood cultures positive for anaerobic gram-negative bacilli and hospitalized for at least 24 hours during the same respective pre- and post-intervention periods were included. Patients were excluded if they were discharged or deceased prior to the pathogen’s genus/species identification, transferred to an out-of-network inpatient facility or institution, received antimicrobial therapy not defined by the Madras-Kelly Antimicrobial Spectrum of Activity Scoring Tool, or were previously included in the study cohort (14).

### Intervention

The laboratory and patient care workflows for pre-intervention and post-intervention groups were used as standard of care during the study periods. Pairs of blood culture bottles (one aerobic and one anaerobic) were inoculated at bedside with patient blood and incubated using BacTec FX (BD, Franklin Lakes, NJ, USA) or Virtuo BacT/ALERT (bioMerieux, Durham, NC, USA). If bacterial growth was detected by the instrument, then a Gram stain was performed. If the Gram stain revealed a single morphology of gram-positive cocci or gram-negative bacilli and the blood culture was the first positive culture within 7 days or yielded a different Gram stain morphology than earlier testing, then molecular testing was used to attempt identification (Verigene gram-negative blood culture test, Luminex/Diasorin, Northbrook, IL, USA). In the pre-intervention group (prior to the implementation of direct from PBCB MALDI-TOF MS identification workflow), gram-negative cocci, gram-positive bacilli, and isolates not identified using Verigene were not attempted to be further identified until after successful subculture on nutrient agar, which typically required at least 48 hours of incubation. After growth on nutrient agar, identification would then be attempted by using MALDI-TOF analysis of an isolated colony. In the post-intervention group, if the Gram stain of a PBCB bottle resulted as gram-negative cocci or gram-positive bacilli, then the PBCB was processed and tested using MALDI-TOF MS. If the Gram stain result was gram-negative bacilli and failed to be identified using Verigene, then PBCB was processed and tested directly using MALDI-TOF MS. The PBCB MALDI-TOF MS testing workflow was implemented on 16 October 2021.

This paragraph describes the PBCB MALDI-TOF MS testing workflow. Bruker updates and maintains the spectral database on the Biotyper system, and a laboratory-modified Sepsityper workflow was used to prepare the PBCB and to analyze the sample. Validation testing of the Bruker RUO database established that a minimum acceptable logarithmic score of 1.70 was required for genus-level identification, and a minimum acceptable logarithmic score of 1.90 was required for species-level identification.

Blood cultures drawn from regional Cleveland Clinic hospitals are couriered to the Main Campus microbiology laboratory every 2 hours. Blood culture bottles are received, tested, and processed 24 hours per day, 7 days per week. PBCB MALDI-TOF testing during the intervention period routinely occurred thrice daily, 7 days per week at approximately 0600, 1400, and 2200.

### Data collection and study definitions

A computer-generated report for the electronic medical health records was used to retrospectively identify all patients with a blood culture positive for an anaerobic gram-negative pathogen during the study period. For the secondary outcome, patients were randomly sampled from the included primary outcome population until the intended sample size of 200 patients was met. Data were retrospectively collected from the electronic medical health records and included patient demographics, microbiology data, antimicrobial data, and clinical outcomes. Comorbidities were collected using ICD-9 and ICD-10 codes.

The primary objective compared the time from Gram stain to pathogen identification in patients with anaerobic gram-negative BSI before and after implementation of the PBCB MALDI-TOF-MS testing workflow. The secondary objective compared the time to antibiotic modification, including escalation and/or de-escalation before and after implementation of the PBCB MALDI-TOF-MS testing workflow.

Time to pathogen identification was determined by the time of Gram stain to the day the blood culture was finalized. MALDI-TOF generally requires pure colonies (single species) for accurate identification. Mixed colonies or mixed growth from a PBCB can interfere, so labs may delay identification until isolation is confirmed. To control for delays in polymicrobial blood cultures, time to anaerobic pathogen identification was assessed at 48 hours.

Time to first antibiotic modification was defined as the time from Gram stain result (time zero) to the time when antibiotic therapy was modified. To objectively determine change in antibiotic therapy, the proposed spectrum-of-activity scoring system from Madras-Kelly was used to identify antimicrobial escalation, de-escalation, or equivalent therapy (14). Escalation of therapy was defined as a change in antimicrobial therapy to one with a higher spectrum of activity score. De-escalation of therapy was defined as a change in antimicrobial therapy to one with a lower spectrum of activity score. For example, when patients present to our institution with suspected infections, they are often started on broad-spectrum antibiotics such as vancomycin and piperacillin-tazobactam (Madras-Kelly score 55.25). Once the causative pathogen is identified, therapy is typically narrowed to ampicillin-sulbactam (Madras-Kelly score 29.50), which is considered a “de-escalation.” Cases where antimicrobial therapy was not modified were excluded from time to antibiotic modification analysis. One-time antibiotic doses and non-antibacterial agents (such as antifungals) were not included in time to antibiotic modification analysis. Categorical data were analyzed using Chi-squared or Fisher’s exact test, as appropriate. Continuous data were analyzed using Mann-Whitney *U* test, as all continuous data were found to be non-normally distributed. Effect estimates with 95% confidence interval (CI) were reported for outcomes and *P*-values were reported for baseline characteristics. Median differences were estimated using quantile regression, and 95% CI for median differences were estimated using bootstrap resampling. Survival analysis was conducted to evaluate the time to pathogen identification. A *P*-value of <0.05 was considered to be statistically significant. Statistical analyses were performed using STATA Statistical Software, version 16.1 (StataCorp, College Station, TX, USA).

## RESULTS

**Fig 1:**
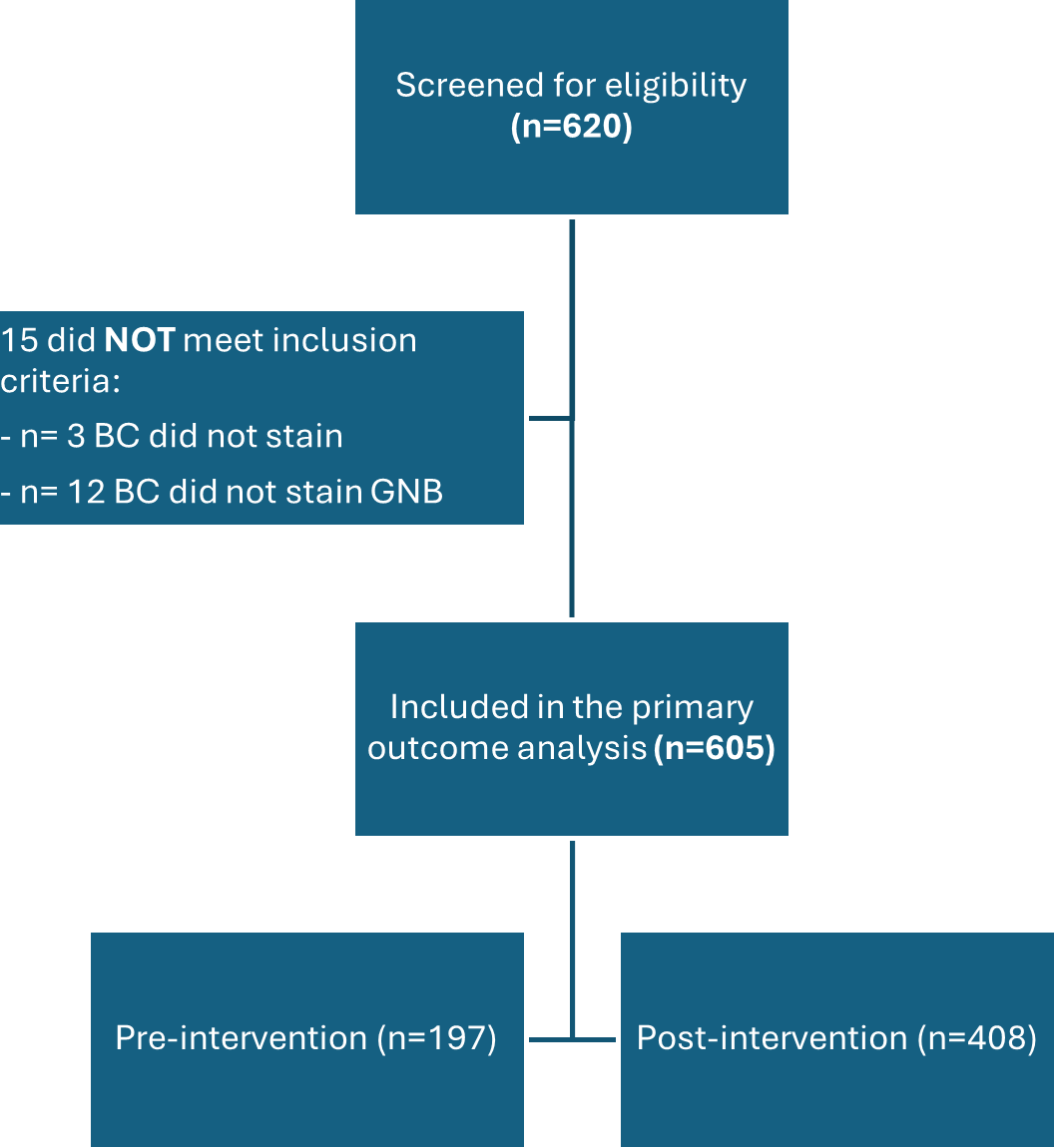
Primary outcome patient selection. BC: blood culture; GNB: gram-negative bacilli.

A total of 620 patients with anaerobic gram-negative BSI were identified during the study period and screened for primary outcome eligibility (Fig. 1). After exclusions, 605 patients were included in the primary outcome analysis, 197 in the pre-intervention group and 408 in the post-intervention group.

**Fig 2:**
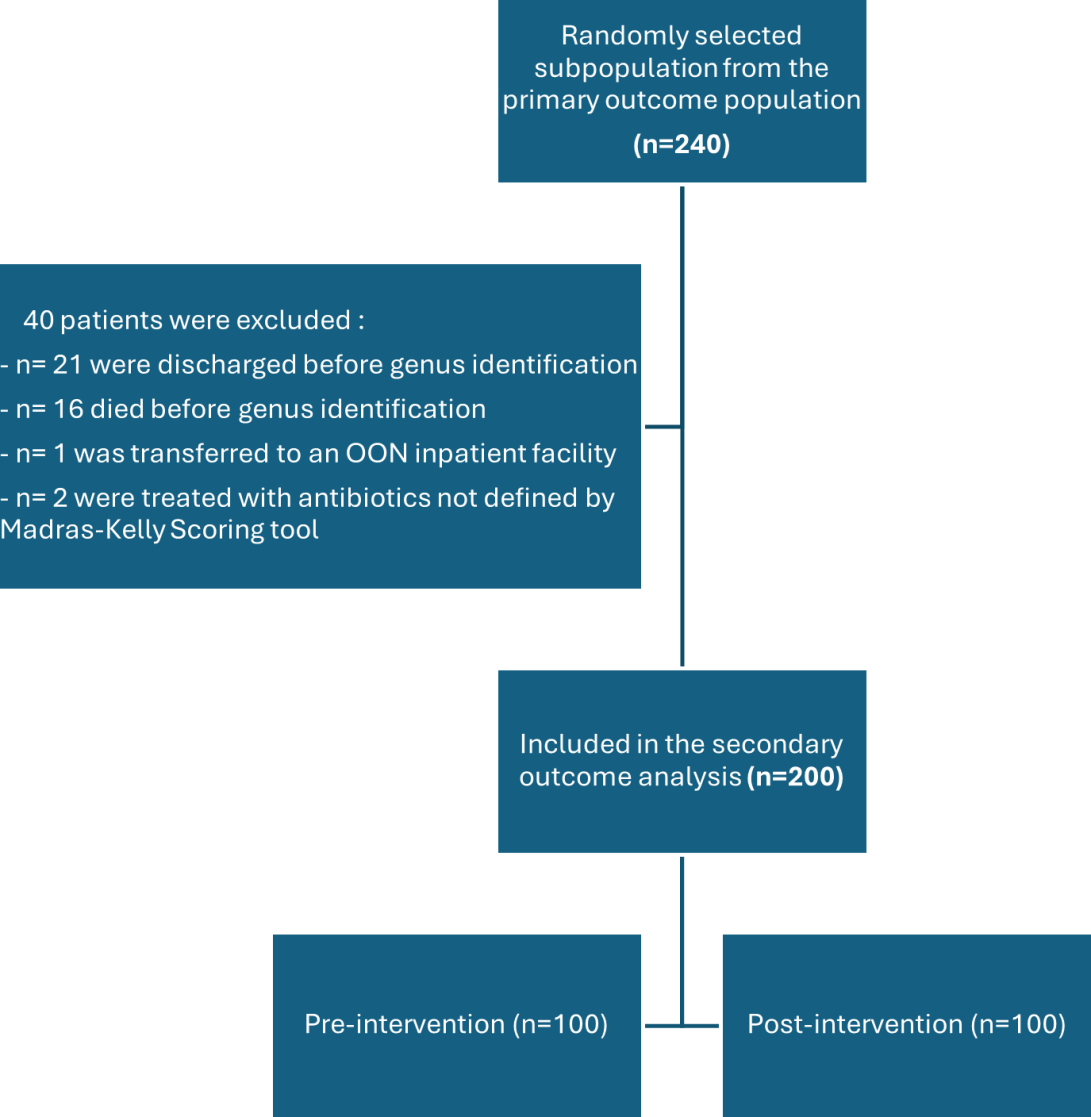
Secondary outcome patient selection. OON: out-of-network.

From the primary outcome population, a random convenience sample of 240 patients was selected as a subpopulation and screened for secondary outcome inclusion (Fig. 2). After exclusions, 200 patients were included in the secondary outcome analysis, 100 in the pre-intervention group and 100 in the post-intervention group.

**Fig 3:**
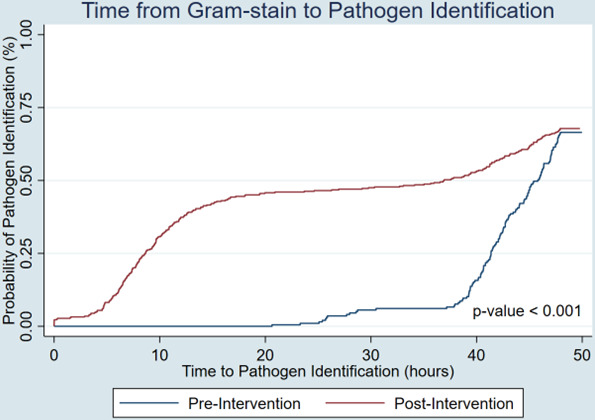
Time to pathogen identification—Kaplan-Meier curve censored to 48 hours.

For the primary outcome (Table 1), median (interquartile range [IQR]) time from Gram stain to pathogen identification was similar in both the pre-intervention and the post-intervention groups (45.8 [41.4–49.7] hours vs 36.7 [8.4–55.2] hours, *P* = 0.15). When the results were censored to 48 hours, time from Gram stain to pathogen identification was significantly lower in the post-intervention group (Fig. 3, *P* < 0.001).

**TABLE 1:** Time to pathogen identification

Outcome	Pre-intervention	Post-intervention	Median difference (95% CI)	*P*-value
Time from Gram stain to pathogen identification, hours; median (IQR)	*n* = 197 45.8 (41.4 to 49.7)	*n* = 408 36.7 (8.4 to 55.2)	−9.0 (−21.7 to 3.2)	0.15
Monomicrobial subgroup analysis				
Time from Gram stain to pathogen identification, hours; median (IQR)	*n* = 148 45.3 (41.5–49.3)	*n* = 254 15.0 (7.8–47.8)	−30.2 (−36.3 to −24.2)	<0.001

A subgroup analysis was performed on monomicrobial blood cultures (Table 1). Time from Gram stain to pathogen identification in monomicrobial blood cultures was significantly lower in the post-intervention group compared to the pre-intervention group (45.3 [41.5–49.3] hours vs 15.0 [7.8–47.8] hours, *P* < 0.001).

Patient demographics were characterized for the secondary outcome subpopulation only (Table 2). The median (IQR) age in the pre-intervention and post-intervention subpopulations was 67 (54–76) years and 72 (63.5–81). More patients in the secondary outcome post-intervention group had a past medical history of chronic kidney disease and required mechanical ventilation at the time of blood culture collection. Twenty-two patients in the pre-intervention group and 47 patients in the post-intervention group had polymicrobial blood cultures. In both groups, the most common pathogen identified was *Bacteroides* species from an intra-abdominal source, as documented on the electronic health record. Most patients in both groups had an infectious disease specialist consult.

**TABLE 2:** Baseline characteristics in subgroup population^*a*^^*,^d^*^

Variable	Pre-intervention (*n* = 100)	Post-intervention (*n* = 100)	*P*-value
Age, years; median (IQR)	67 (54–76)	72 (63.5–81)	0.01
Gender, male	43 (43)	55 (55)	0.09
Comorbidities			
CKD	30 (30)	44 (44)	0.04
DM	37 (37)	33 (33)	0.55
SOT	0 (0)	1 (1)	1.0
BMT	2 (2)	0 (0)	0.5
Vasopressor use^*b*^	4 (4)	10 (10)	0.1
Mechanically ventilated^*b*^	2 (2)	10 (10)	0.02
Renal replacement therapy^*b*^	3 (3)	0 (0)	0.25
COVID positive during admission	5 (5)	5 (5)	
Chronic use of antibiotics prior to admission	4 (4)	5 (5)	1.0
Blood culture collection location			0.19
Large hospital	67 (67)	58 (58)	
Community hospital	33 (33)	42 (42)	
Non-ICU admission	68 (68)	64 (64)	0.55
Polymicrobial blood cultures	22 (22)	47 (47)	<0.001
BSI source			0.48
Catheter-related/foreign material	4 (4)	3 (3)	
Genitourinary	6 (6)	8 (8)	
Intra-abdominal	62 (62)	52 (52)	
Other	20 (20)	26 (26)	
Respiratory tract	0 (0)	3 (3)	
Unknown	8 (8)	9 (9)	
Pathogen identified			<0.001
*Bacteroides* spp.	100 (100)	84 (84)	
*Biophilia* spp.	0 (0)	1 (1)	
*Fusobacterium* spp.	0 (0)	12 (12)	
*Parabacteroides* spp.	0 (0)	3 (3)	
Infectious disease consult	85 (85)	90 (90)	0.29
Number of antibiotic modifications			0.41
0	13 (13)	6 (6)	
1	29 (29)	32 (32)	
2	30 (30)	33 (33)	
≥3	28 (28)	29 (29)	
First antimicrobial modification			0.02
Escalation	35 (35)	25 (25)	
De-escalation	39 (39)	60 (60)	
30-day all-cause mortality	15 (15)	20 (20)	0.35
Discharge disposition			0.11
Outpatient facility^*c*^	37 (37)	28 (28)	
Against medical advice discharge	1 (1)	0 (0)	
Expired	8 (8)	10 (10)	
Home	49 (49)	47 (47)	
Hospice	5 (5)	15 (15)	
Hospital LOS, days; median (IQR)	8.2 (5.3–12.6)	8.0 (5.1–14.0)	0.85

^*a*^ Reported as no. (%) unless otherwise noted.

^*b*^ Vasopressor use, mechanical ventilation, and renal replacement therapy were assessed at the time of blood culture collection.

^*c*^ Outpatient facilities include long-term acute care facilities, acute rehab, skilled nursing home facilities or assisted living facilities.

^*d*^ CKD, chronic kidney disease; DM, diabetes mellitus; SOT, solid organ transplant; BMT, bone marrow transplant; ICU, intensive care unit.

Time to escalation and time to de-escalation of antibiotic therapy were similar in both the pre-intervention and post-intervention groups (Table 3). The analysis of monomicrobial blood cultures only (*n* = 54 in pre-intervention and *n* = 41 in post-intervention) demonstrated a significant decrease in median (IQR) time to antibiotic de-escalation in the post-intervention group vs the pre-intervention group (52.0 [24.0–64.5] hours vs 21.1 [7.9–51.6] hours, *P* = 0.014) (Table 3).

**TABLE 3:** Time to antibiotic modification

Outcome	Pre-intervention	Post-intervention	Median difference (95% CI)	*P*-value
Time to first antibiotic escalation, hours; median (IQR)	*n* = 35 13.2 (3.7 to 39.9)	*n* = 25 24.4 (13.8 to 91.3)	11.2 (−19.6 to 42.1)	0.47
Time to first antibiotic de-escalation, hours; median (IQR)	*n* = 39 45.1 (19.8 to 62.7)	*n* = 60 28.0 (10.5 to 49.7)	−17.0 (−38.0 to 4.0)	0.11
Monomicrobial subgroup analysis				
Time to first antibiotic escalation, hours; median (IQR)	*n* = 25 14.5 (3.7 to 35.9)	*n* = 12 29.6 (10.3 to 54.4)	20.3 (−9.0 to 49.5)	0.17
Time to first antibiotic de-escalation, hours; median (IQR)	*n* = 29 52.0 (24.0 to 64.5)	*n* = 29 21.1 (7.9 to 51.6)	−30.9 (−55.2 to −6.6)	0.01

## DISCUSSION

No prior studies, to our knowledge, have addressed the impact of direct-from-PBCB MALDI-TOF MS use for rapid anaerobic gram-negative pathogen identification and its impact on antibiotic therapy modifications. In this study, the use of direct-from-PBCB MALDI-TOF significantly reduced time to pathogen identification and time to antibiotic de-escalation in monomicrobial anaerobic gram-negative bacteremia.

Previous studies have shown that MALDI-TOF MS decreases time to pathogen identification by up to 24 hours compared with conventional diagnostics on a broader scale (15). Perez et al. (16) tested the hypothesis of whether patient care would be enhanced before and after combining new rapid pathogen identification with MALDI-TOF MS from agar subcultures coupled with antimicrobial stewardship program (ASP) for patients with gram-negative BSI. It is important to note that at the time this study was conducted, MALDI-TOF MS use for identification of anaerobic gram-negative organisms directly from PBCB had not been validated. This study reported that the integration of rapid identification and susceptibility techniques with antimicrobial stewardship significantly improved time to optimal therapy. The mean time to gram-negative organism identification was significantly longer in the pre-intervention group vs the intervention group (36.6 ± 15.3 hours vs. 11.1 ± 10.2 hours, respectively; *P* < 0.001). The average time to initiation of an active agent with *in vitro* susceptibility against the target pathogen was 73.2 hours in the pre-intervention group compared with 36.5 hours in the intervention group (*P* < 0.001). Even though this study did not evaluate the appropriateness of antibiotic therapy, findings align similarly with our study. The study conducted by Perez et al. demonstrated a benefit in optimizing antimicrobial therapy when ASP was coupled with the use of RDTs.

Although not evaluated in our study, pharmacist-led ASP at CCHS has previously demonstrated positive impacts on antimicrobial therapy, including decreased time to antimicrobial switch and time to effective therapy, consistent with findings reported by Perez et al., Neuner et al. and Rivard et al. (16–18). At CCHS, when a pathogen is identified and documented in the electronic health record, a real-time notification is sent to the antimicrobial stewardship in-basket. This process enables an infectious diseases-trained pharmacist to promptly review the culture results and current antimicrobial regimen. If optimization is warranted, the pharmacist provides targeted recommendations to the primary team. Neuner et al. (17) demonstrated that the combination of RDT (Verigene BC-GP) coupled with ASP intervention decreased time to antimicrobial switch (75 ± 46 hours vs. 48 ± 41 hours; *P* < 0.001) and time to antimicrobial de-escalation (82 ± 48 hours vs 53 ± 41 hours; *P* < 0.001). Similarly, Rivard et al. (18) evaluated the effect on time from Gram stain to antimicrobial modification of the Nanosphere Verigene aerobic gram-negative blood culture test (BC-GN) paired with an active ASP at CCHS. The median time from Gram stain to antimicrobial switch was significantly decreased in the post-intervention group vs the pre-intervention group (28.6 vs.44.1 hours, respectively; *P* = 0.004). The authors concluded that rapid microarray testing on blood cultures with active antimicrobial stewardship intervention was associated with decreased time to antimicrobial switch and time to effective therapy.

It is worth noting that certain molecular RDTs, such as the bioMérieux BioFire BCID2 and the Roche (GenMark) ePlex BCID Panel, can detect *Bacteroides* spp. As such, these RDTs may offer a similar clinical benefit to direct-from PBCB MALDI-TOF MS in terms of early organism identification, particularly in the context of anaerobic gram-negative blood cultures (19, 20). Their incorporation into diagnostic algorithms should be considered as a viable alternative, depending on institutional resources and workflow integration.

While the current study did not evaluate the impact of rapid anaerobic gram-negative pathogen identification on patient clinical outcomes, such as mortality and hospital LOS, and the effect of antimicrobial stewardship coupled with rapid pathogen identification, many studies have evaluated these outcomes on gram-negative BSIs. In those studies, empiric treatment prior to blood culture identification was inadequate in up to 58% of patients, therefore increasing all-cause mortality and hospital LOS (21–23). Rapid pathogen identification has been correlated with a reduction in time to initiation of appropriate antimicrobial therapy, particularly when integrated with ASPs, ultimately contributing to improved patient survival (24).

The limitations of this study must be considered. First, this was a retrospective study with a small sample size. While bootstrap resampling is a powerful statistical technique used when sample sizes are small, it relies on the assumption that the original sample is representative of the underlying population. The bootstrapped estimates may reflect the specific microbiological and clinical context of our institution, limiting their generalizability to other settings with different patient populations or diagnostic workflows. Furthermore, the differences in baseline characteristics could not be adjusted for confounding variables. The baseline characteristics demonstrated that the post-intervention patient population may have been sicker compared to the pre-intervention population. We did not adjust for any variables as potential confounders because they did not meet the criteria of true confounders—specifically, they were not independently associated with both the exposure (e.g., MALDI-TOF-MS implementation) and the outcome (e.g., antibiotic modification) in a way that would bias the observed relationship. Including such variables in the analysis could introduce overadjustment bias or obscure the true effect of the intervention, rather than clarify it. The scoring tool also did not account for intravenous to oral antibiotic switches. However, the scoring tool was validated to identify de-escalation in antimicrobial therapy, with a sensitivity and specificity of 86.3% and 96.0% compared to expert opinion (14). We also did not include any one-time antibiotic therapy doses given to the patient when assessing spectrum-of-activity and antibiotic modification, as these are often administered as broad-spectrum coverage upon initial presentation or clinical deterioration and may be misclassified as de-escalation; instead, antibiotics were evaluated starting from the time of Gram stain results to more accurately capture therapy changes influenced by the pathogen identification method utilized in each group.

In conclusion, rapid anaerobic gram-negative pathogen identification using MALDI-TOF MS directly from PBCB significantly reduced time to pathogen identification, being more pronounced in the monomicrobial subgroup, as well as time to antibiotic therapy de-escalation. These results support the use of rapid pathogen identification methods for patients with anaerobic gram-negative BSIs.
